# Size-resolved emission rates of airborne bacteria and fungi in an occupied classroom

**DOI:** 10.1111/j.1600-0668.2012.00769.x

**Published:** 2012-08

**Authors:** J Qian, D Hospodsky, N Yamamoto, W W Nazaroff, J Peccia

**Affiliations:** 1 Department of Chemical and Environmental Engineering, Yale UniversityNew Haven, CT, USA; 2 Department of Civil and Environmental Engineering, Clarkson UniversityPotsdam, NY, USA; 3 Japan Society for the Promotion of ScienceIchiban-cho 8, Chiyoda-ku, Tokyo, Japan; 4 Department of Civil and Environmental Engineering, University of CaliforniaBerkeley, CA, USA

**Keywords:** Bioaerosols, Resuspension, qPCR, Particle size distribution, Indoor microbiome, 454 pyrosequencing, Human microbiome

## Abstract

The role of human occupancy as a source of indoor biological aerosols is poorly understood. Size-resolved concentrations of total and biological particles in indoor air were quantified in a classroom under occupied and vacant conditions. Per-occupant emission rates were estimated through a mass-balance modeling approach, and the microbial diversity of indoor and outdoor air during occupancy was determined via rDNA gene sequence analysis. Significant increases of total particle mass and bacterial genome concentrations were observed during the occupied period compared to the vacant case. These increases varied in magnitude with the particle size and ranged from 3 to 68 times for total mass, 12–2700 times for bacterial genomes, and 1.5–5.2 times for fungal genomes. Emission rates per person-hour because of occupancy were 31 mg, 37 × 10^6^ genome copies, and 7.3 × 10^6^ genome copies for total particle mass, bacteria, and fungi, respectively. Of the bacterial emissions, ∼18% are from taxa that are closely associated with the human skin microbiome. This analysis provides size-resolved, per person-hour emission rates for these biological particles and illustrates the extent to which being in an occupied room results in exposure to bacteria that are associated with previous or current human occupants.

**Practical Implications:**

Presented here are the first size-resolved, per person emission rate estimates of bacterial and fungal genomes for a common occupied indoor space. The marked differences observed between total particle and bacterial size distributions suggest that size-dependent aerosol models that use total particles as a surrogate for microbial particles incorrectly assess the fate of and human exposure to airborne bacteria. The strong signal of human microbiota in airborne particulate matter in an occupied setting demonstrates that the aerosol route can be a source of exposure to microorganisms emitted from the skin, hair, nostrils, and mouths of other occupants.

## Introduction

Modeling the dynamics of airborne particulate matter is a powerful tool for understanding how building design, occupancy, and operation affect human exposure to airborne particles (Nazaroff and Cass, 1989). Key physical processes affecting indoor particles such as penetration, deposition, resuspension, filtration, and indoor emissions can each vary strongly with particle size (Hinds, 1982; Liu and Nazaroff, 2001; Nazaroff, 2004; Oberdörster et al., 2005; Riley et al., 2002; Thatcher and Layton, 1995). Size-dependent particle behavior often can be associated with specific chemical and biological constituents of particulate matter provided that the particle size distribution of the constituent is known. Due significantly to limitations of culture-based sampling and analysis that have constrained efforts to study the full microbial ecology of air (Amann et al., 1995; Pace, 1997; Peccia and Hernandez, 2006), modeling tools for simulating indoor aerosols have not been systematically applied to the biological components of particulate matter. As a consequence, our knowledge of microbes in indoor air lags behind that of total particulate matter and its chemical constituents, even though the impacts of biological aerosol exposure on human health and well-being are considerable (D'Amato et al., 2005; Monto, 2002; Pope et al., 1993; WHO, 1999).

One important knowledge gap is in understanding how human occupancy results in the emission of bacterial and fungal particles into indoor air. Observational and mechanistic studies have demonstrated the importance of human occupancy as a source of total aerosol mass (Ferro et al., 2004a, b; Koistinen et al., 2004; Thatcher and Layton, 1995). More specifically, in the absence of combustion, cooking, and smoking, evidence indicates that resuspension can be a major source of total airborne particulate matter in occupied indoor environments, thereby suggesting a potentially important emission mechanism for indoor biological particles. In addition, direct human emissions such as skin shedding (Noble et al., 1976), talking, coughing, and sneezing (Morawska, 2006) may play a significant, but less well-characterized role influencing the content and character of indoor microbiological aerosols. Investigators have previously noted both the significant content of desquamated human skin cells in aerosols in occupied settings (Clark, 1974; Fox et al., 2008; Noble et al., 1976), as well as the presence of bacteria, including *Staphylococcus*, *Propionibacteria*, *Corynebacteria*, and enteric bacteria, that are typically ascribed to human microflora (Bouillard et al., 2005; Fox et al., 2010; Noris et al., 2011; Rintala et al., 2008; Täubel et al., 2009). However, the size-resolved emission rates of bacteria and fungi due to human occupancy of indoor environments have not been reported, which limits modeling efforts to predict the fate and transport of these important components of particulate matter.

The goal of this research is to determine the human-associated emission rates of bacteria and fungi in an occupied classroom. Particle size distributions of total airborne particulate matter, bacterial genomes, and fungal genomes (based on rRNA-encoding gene copy numbers) were measured under occupied and vacant conditions, and a material balance model was applied to determine the per person emission rates of bacterial and fungal size-fractionated particles attributable to occupancy. Bacterial phylogenetic libraries were produced for aerosols sampled under occupied conditions, and the size-resolved emissions of human-associated bacterial taxa were estimated. The size-resolved emission rates and bacterial population characterization produced here represent an important step toward applying models to make inferences about the distribution of and human exposure to bacteria and fungi in occupied indoor settings.

## Materials and methods

### Test environment

A university classroom located on the ground floor of a five-story building in the northeastern United States was selected as the test room. This location was selected because of consistent and readily characterized occupancy levels, along with proximity to the research team and relative ease of securing access. Experiments were conducted in the fall of 2009 and included 4 days when the classroom and building were occupied, and 4 days when the classroom and building were vacant. On occupied days, the room was frequently accessed with three to five classes per weekday. For the four test days when the room was occupied, the room held an average of 4.7 people during a total of 22.2 h of sampling for a cumulative occupancy of 104.2 person hours. Classes were conducted in a lecture format. Students typically sat in desks and the instructor stood or moved throughout the front half of the classroom. Although the floor is vacuumed regularly, no cleaning was conducted at least 1 day before or during the experimental days. The volume of the classroom is approximately 90 m^3^ (*L* = 6.0 m, *W* = 5.0 m, *H* = 3.0 m), and the entire floor area was covered by lightly worn commercial medium pile level-loop carpet. No mold or moisture problems have been reported for this building, and none have been observed during sampling. The outdoor environment near the building was highly vegetated and consisted of a tree-lined street with lawn and flower gardens. There were no green plants in the room, nor were they common throughout the building.

During the occupied and vacant experiments, all windows and doors were closed and conditioned air was delivered by the building HVAC system to the room through a 0.9 × 0.05 m air register situated above the door. Ventilation exhaust ports were located along the floor and near the wall opposite to the ventilation air inlet. Based on six deliberate CO_2_ releases and tracer gas studies performed prior to air sampling and under vacant, well-mixed conditions, the average air-exchange rate (AER) ± s.d. was 5.5 ± 1.3/h. AER was also calculated immediately after occupancy based on the decay of exhaled CO_2_ from occupants. This AER averaged 6.2 ± 0.9/h, which is not statistically different than the AER derived from tracer gas studies. During the four occupied and four vacant experimental days, the temperature was 23.5 ± 1.1°C indoors and 13.4 ± 2.4°C outdoors, and the average relative humidity was 28 ± 7% indoors and 45 ± 16% outdoors. The reported air-exchange rate is in the upper range for ventilated commercial buildings (Persily et al., 2006). The average occupant density of 4.7 persons in 30 m^2^ (16 persons per 100 m^2^) is within ranges common for many indoor spaces with moderately dense occupancy (ASHRAE, 2005).

### Sampling

Both indoor and outdoor aerosols were sampled during the four occupied and four unoccupied experimental days. For indoor and outdoor size-distributed samples, particles were collected on uncoated polycarbonate track-etched filters (PCTE) that were loaded onto an 8-stage non-viable impactor (New Star Environmental Inc., Roswell, GA, USA) operated at a flow rate of 28.3 l/min. To increase the mass of collected material, six stages of the impactor were used with the following nominal cut-points: 9.0, 4.7, 3.3, 2.1, 1.1, and 0.4 μm. An upper cut-point for the initial stage was assumed to be 20 μm, based on the rapid removal of particles greater than 20 μm from indoor air owing to gravitational settling. To obtain genome copies above detection levels (Hospodsky et al., 2010) on all stages, the non-viable impactors sampled air cumulatively for the four consecutive occupied or vacant experimental days. Between experimental days, the impactors were wrapped in autoclaved aluminum foil and stored at 4°C to inhibit bacterial and fungal growth. In addition to these impactor samplers, semi-continuous particle counts were recorded indoors and outdoors on occupied and vacant days using optical particle counters with the following size channels: 0.3–0.5, 0.5–1.0, 1.0–2.5, 2.5–5.0, and 5.0–10.0 μm (MET ONE HHPC-6, Hach Ultra Analytics Inc., Loveland, CO, USA).

### Quantification of size-resolved particle mass, bacterial genome copies, and fungal genome copies

To determine particulate matter mass concentrations from impactors, filters were equilibrated and weighed before and after sampling. Weighing was performed using a precision balance (Mettler Toledo type AG245, Columbus, OH, USA). Static electricity was removed with a polonium αparticle source (Staticmaster static eliminator, NRD, Grand Island, NY, USA), and filters were equilibrated prior to weighing at constant temperature and humidity (30 ± 0.5°C, 32 ± 1.5% RH) for at least 24 h before weighing. Weighing was performed in triplicate and averages reported.

The quantification of bacterial 16S rRNA-encoding genes and fungal 18S rRNA-encoding genes in all impactor stages was completed using real-time PCR in accordance with previously developed protocols for DNA extraction, amplification, and calibration (Boreson et al., 2004; Hospodsky et al., 2010). Briefly, DNA was extracted from one-quarter of the PCTE filter using a method that included cell lysis by enzymatic treatment and physical disruption through bead beating, phenol/chloroform isoamyl alcohol-based isolation of nucleic acids, and DNA cleanup and concentration using spin columns from the MO BIO PowerMax Soil DNA kit (MO BIO, Inc., Carlsbad, CA, USA) (Boreson et al., 2004). Exceptions to the cited method included proteinase K incubation at 54°C instead of 37°C, omitting the freeze–thawing cycle during DNA extraction, and omitting the 1-h, 65°C incubation step prior to beadbeating. Quantitative PCR was performed in triplicate using an ABI 7500 fast real-time PCR system (Applied Biosystems, Carlsbad, CA, USA). For bacteria, universal bacterial primers and TaqMan^**®**^ probes covered the 331 to 797 *E. coli* numbering region of the 16S rRNA-encoding gene with forward primer 5′-TCCTACGGGAGGCAGCAGT-3′, reverse primer 5′-GGACTACCAGGGTATCTAATCCTGTT-3′, and probe (6-FAM)-5′-CGTATTACCGCGGCTGCTGGCAC-3′-(BHQ-1) (Nadkarni et al., 2002). For the universal fungal DNA quantification, a SybrGreen assay was used with primers reported by Zhou et al. (2000). These included the forward primers FF2 (5′-GGTTCTATTTTGTTGGTTTCTA-3′) and reverse primer FR1 (5′-CTCTCAATCTGTCAATCCTTATT-3′). Real-time PCR standard curves of genome quantity vs. cycle threshold number for bacteria and fungi were developed using known amounts of *Bacillus atrophaeus* (ATCC 49337) and *Aspergillus fumigatus* (ATCC 34506) genomic DNA, respectively. To produce standard curves, three to five independent dilution series were produced corresponding to 10^1^ to 10^6^ genome copies for each organism. For bacteria, cycle threshold values were calibrated vs. total bacterial genomes and accounted for the ten rRNA operon copies in *B. atrophaeus* and the average of four rRNA operon copies per genome for all bacteria (Lee et al., 2009). The rRNA operon copy numbers in fungi are highly variable, even within species, and are not well cataloged. Thus, fungal universal qPCR values are presented as *A. fumigatus* genome equivalents by assuming 55 rRNA operon copies per genome for *A. fumigatus* (Herrera et al., 2009). To test for PCR matrix inhibition, standard curves for *B. atrophaeus* and *A. fumigatus* were also produced by spiking genomes into aerosol filter extract. No inhibitory effects were observed.

### Emission rate estimation

Total and microbial particle emission rates during occupancy were estimated by considering the room as a completely mixed flow-through reactor in which the time-averaged indoor concentration is the sum of two contributions: a fraction, *f*, of the time-averaged outdoor concentration, plus a contribution from indoor emissions:


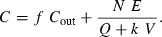
(1)

In Equation (1), *C* is the time-averaged indoor air concentration of total particle mass (μg/m^3^), bacterial genomes (genomes/m^3^), or fungal genomes (equivalent genomes/m^3^); *C*_out_ is the time-averaged corresponding outdoor concentration during monitoring, *f* is the indoor–outdoor ratio in the absence of indoor emissions (measured during the vacant sampling period); *N* is the average number of persons in the room during the occupied experimental time (persons); *E* is the per person emission rate of total particle mass (μg/h/person), bacterial genomes (genomes/h/person) or fungal genomes (equivalent genomes/h/person), *Q* is the volumetric ventilation rate (m^3^/h); *V* is the room volume (m^3^), and *k* is the size-specific deposition-rate coefficient for total particles, bacterial genomes, or equivalent fungal genomes (per h). The ventilation rate was estimated as the product of the room volume and the measured air-exchange rate. Size-specific deposition-rate coefficients were derived from previously reported values (Thatcher et al., 2002) and are listed in Table 1. These specific rates were chosen because of their coverage of the super micron range (for 0.5–9 μm particles) and were extrapolated to the largest size section considered in the present work. Utilizing the impactor-measured indoor and outdoor concentrations for the occupied and vacant sampling periods, Equation (1) was solved for the per person, size-resolved emission rates (*E*) corresponding to total airborne particle mass, bacterial genomes, and equivalent fungal genomes.

**Table 1:** Deposition-rate coefficients (*k*) for each impactor size stage used to estimate particle mass, bacterial genome, and equivalent fungal genome emission rates^a^

	0.4–1.1 μm	1.1–2.1 μm	2.1–3.3 μm	3.3–4.7 μm	4.7–9 μm	>9 μm
Deposition loss-rate coefficient (/h)	0.31	0.79	2.1	4.4	8.6	9.6

a Rate coefficients based on experimental deposition data for an unoccupied room reported by Thatcher et al. (2002). We used data from Table 2 in that paper, for high average airspeed (19.1 cm/s) and for furnished conditions. Our approach entailed fitting a cubic equation to the tabulated log of particle diameter vs. the log of loss-rate coefficient. We then computed the average loss rate for each impactor size bin utilizing this cubic equation and assuming that the particle mass was uniformly distributed with respect to log of particle diameter within each size section. The cubic equation is *Y* = −0.407 + 1.232*X* + 1.818*X*^2^−1.594*X*^3^, where *Y* = log(*k*) and *X* = log(*d*_p_), *k* is the deposition loss-rate coefficient in per hour units, and *d*_p_ is the particle aerodynamic diameter in μm units. The average absolute error between the cubic equation and the tabulated data in Thatcher et al. (2002) is 4%.

### Generation of phylogenetic libraries and data analysis

Phylogenetic library preparation was conducted for a total of 11 samples that included the five stages of the indoor occupied impactor sampler and six stages for the outdoor occupied impactor. Stage 2.1–3.3 μm of the indoor impactor had no detectable PCR amplification and was thus excluding from library preparation. Libraries of bacteria were produced by pyrosequencing on a GS FLX sequencer, which utilized the Titanium series chemistry (454 Life Sciences, Branford, CT, USA). PCR primers for bacteria covered the 343F to 926R (Liu et al., 2007; Wang and Qian, 2009) region of the 16S rRNA-encoding gene and also included pyrosequencing adaptors, keys, and multiplex identifiers. Four PCRs were conducted for each sample, and amplicons were combined before removing salts and unincorporated primers using a Qiagen MinElute PCR purification kit (Qiagen Inc., Valencia, CA, USA) (Bibby et al., 2010). Amplicons were visualized on a 1.2% agarose gel and if smearing was observed, amplicons were extracted directly from the gel using a Qiagen MinElute gel extraction kit (Qiagen Inc.). Sequencing was performed at the Yale Center for Genome Analysis.

Quantitative sequence analysis was performed using tools in the Quantitative Insights Into Microbial Ecology (QIIME) tool box (Caporaso et al., 2010). Within QIIME, sequences were sorted by their multiplex identifier, and primer and multiplex identifier sequences were trimmed. Sequences were excluded if they could not be sorted by multiplex identifier, contained an undefined base, or were shorter than 200 nucleotides. Similar sequences were clustered into operational taxanomic units based on 97% similarity, aligned with the Greengenes core set (DeSantis et al., 2006), and taxonomy was assigned using the Ribosomal Database Project (RDP) classifier (Cole et al., 2009). The unprocessed DNA sequences obtained in this study have been deposited in the MG-RAST (metagenomics.anl.gov) (Meyer et al., 2008) archive under accession number 4470369.3.

## Results

### Particle size distribution

**Fig. 1:**
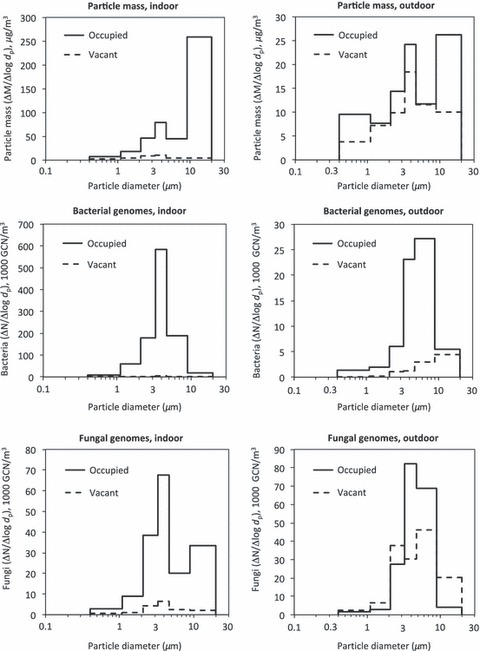
Size distributions of total particle mass, bacterial genomes, and equivalent fungal genome concentrations for indoor and outdoor aerosols collected under occupied and vacant states in a university classroom. GCN denotes genome copy number. An upper limit of 20 μm is assumed for the largest impactor stage

Figure 1 shows the size distributions of indoor and outdoor particle mass and the bacterial and fungal genome copies obtained from the impactor measurements under occupied and vacant conditions. These data reveal important insights regarding concentrations and size distributions that result from room occupancy. First, human occupancy was associated with substantially increased airborne concentrations of total particles, bacterial genomes, and equivalent fungal genomes. For each measured size range, the indoor concentrations are higher when the room is occupied compared with being vacant (paired *t*-test, *P* < 0.05 for bacteria and fungi). Averaged across all size ranges, the indoor air occupied-to-vacant ratios of particulate matter mass concentrations, bacterial genome concentrations, and equivalent fungal genome concentrations were 15, 170, and 2.3, respectively. Only minor differences were observed between the outdoor levels when comparing occupied and vacant sampling days, which indicates that the increases in the indoor concentrations during occupancy were not caused by concomitant changes in outdoor levels. In addition, during vacant conditions, indoor size-distributed concentrations of particulate matter, bacterial genome, and equivalent fungal genome concentrations were qualitatively similar to those measured outdoors (Figure 1).

In support of these observations from filter-based sampling, the real-time optical particle counter data reinforce the finding that occupancy is a major contributor to supermicron particle concentrations. Comparison of Figures S1–S4 (vacant sampling days) to Figures S5–S8 (occupied sampling days) in the online supporting information demonstrates the substantial increases in super micron particle concentrations when the room is occupied. The estimated mass-based particle size distributions obtained from analysis of the OPC data (Figure S9) reveal a profile that is similar to the filter-based measurements (Figure 1) with respect to total concentration and the mass distribution being weighed toward the largest particles.

Examining the particle size distributions, Figure 1 shows that the increase in total particle mass during occupancy is dominated by the largest measured supermicron particles, that is those greater than 9 μm in aerodynamic diameter. The distribution of fungal genome copies was somewhat different than that of total particulate matter, with fungal peaks during occupancy near the typically cited aerodynamic diameters of unicellular (2–5 μm) and multicellular fungal spores (>10 μm) (Eduard, 2009; Jankowska et al., 2000; Lee et al., 2005; McCartney et al., 1993; Reponen, 1995). The largest multiplicative increase in airborne equivalent fungal genome concentrations associated with occupancy was observed in the largest (>9 μm) stage. In contrast, for bacterial genomes, a strong peak during occupancy was observed on the stage corresponding to the 3–5 μm aerodynamic diameter range.

**Fig. 2:**
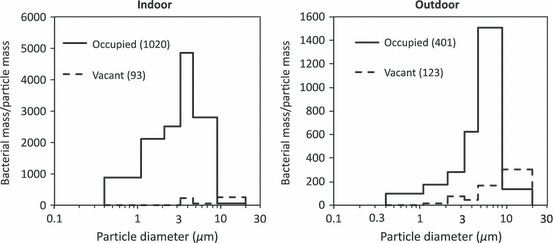
Size-resolved bacterial content of total aerosol mass. Values represent the ratio (in parts per million, ppm) of bacterial mass to total aerosol mass for each impactor stage. The bacterial proportions (in ppm) for the entire size range measured are presented in the figure legend in parentheses

Figure 2 plots the ratio of estimated bacterial mass to particulate matter mass for all the measured size ranges. The results demonstrate that indoor airborne particulate matter during occupancy was enriched in bacteria, especially in the 3–5 μm range, and that the contribution of bacteria to particulate matter mass was about 0.5% (5000 ppm) in this size range. Overall, bacteria contributed approximately 0.1% (1000 ppm) to indoor airborne particle mass during occupancy. In these calculations, bacterial mass was estimated assuming the weight of a bacterium to be 655 femtograms (Ilic et al., 2001). An analogous mass estimate was not conducted for fungi owing to the known variability of 18S rDNA gene copy numbers per genome among different fungal species and the large variability in the size (and mass) of unicellular and multicellular spores.

### Emission rates

The material balance model applied to determine emission rates assumes that within a specific size range, the concentration of a species in indoor air can be estimated as a fraction of the level in outdoor air, plus the contribution from indoor emissions. The contribution from the indoor source is estimated from a balance between the emission rate (associated with occupancy) and the rates of removal (by ventilation plus deposition). To determine the size-specific infiltration factor, *f*, we analyzed the indoor–outdoor ratio for each size range during vacant conditions and assumed that the same ratios apply for each respective size in the occupied case. Also, during spans of time on the occupied monitoring days when the classroom was vacant (e.g., during breaks between classes), relatively consistent relationships were observed between the indoor and outdoor particle number concentrations for finer particles. During these same unoccupied intervals, the OPC data also demonstrate that indoor concentrations of supermicron particles are small (see Figures S5–S8).

**Fig. 3:**
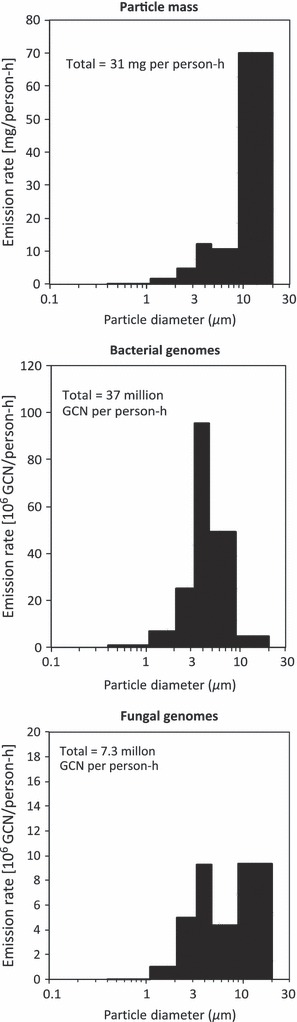
Per person emission rates of total particles, bacterial genomes, and equivalent fungal genomes in an occupied university classroom. Average human occupancy during these experiments was 4.7 persons. Emission values on the *y*-axis are presented as (Δ*E*/Δlog *d*_p_)

The estimated, per person, size-resolved emission rates of particulate matter mass, bacterial genomes, and equivalent fungal genomes attributable to human occupancy are presented in Figure 3. The size-distributed emission rates are similar in shape to the time-averaged concentrations during occupied periods. The majority of emissions for total mass are associated with particles larger than 9 μm, whereas bacterial genome emissions are predominantly in the 3–5 μm aerodynamic diameter range.

It is important to acknowledge that these rates represent aggregate emissions associated with human occupancy, potentially including contributions from resuspension from the carpeted floor and from other surfaces plus direct shedding of microorganisms and particles from humans. The nature of these experiments does not allow us to apportion occupancy-associated emissions into their separate components for total particulate matter. However, phylogenetic analysis does provide some clues as to the origin of airborne bacterial genomes, as described next.

### Bacterial population analysis

**Fig. 4:**
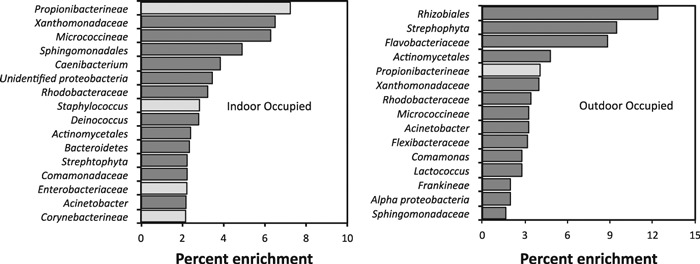
Bar chart demonstrates the relative abundances of the 15 most common bacterial taxa in the indoor occupied and outdoor occupied samples. Groups are classified to the highest taxonomic level to which they could be confidently assigned. Groups shown represent 57% of the occupied indoor air taxa and 68% of the outdoor air taxa. Those taxa that are associated with the human microbiome are presented in light gray

For the 11 impactor samples considered, a total of 621 sequence reads with an average length of 462 nucleotides were generated after trimming and quality control. Phylogenetic data are presented in Figure 4 while abundance estimates (phylum level) averaged across all size ranges are included in the online supplemental information (Figure S10). Figure 4 allows for comparison of the most abundant taxa in indoor air during occupied conditions vs. outdoor air under occupied conditions. The indoor and outdoor air share many similarities in the taxa including the presence of *Sphingomonas*, *Rhodobacteria*, and *Streptophyta* (chloroplasts from green plants), each of which is common in outdoor bioaerosols and in the outdoor environment (Bowers et al., 2011; Brodie et al., 2007; Lighthart, 1997). The presence of these outdoor taxa indoors suggests that indoor air is partly comprised of outdoor air and resuspended dust (that may have had an outdoor origin). However, in addition, the indoor occupied aerosol microbial ecology shows a distinctive signature of human skin microflora. Dominant taxa for human-associated bacteria are becoming well described for human skin, hair, nostrils, and the oral cavity. Specific taxa within these populations that are also not commonly found in the outdoor environment include members of the genera *Staphylococcus*, *Corynebacteria*, and *Proprionibacteria*. Members of the genera *Staphylococcus*, *Prevotella*, *Fusobacterium*, and *Veilionella*, and *Pasteurella* make up the majority of unique organisms isolated from the oral cavity (Costello et al., 2009; Grice and Segre, 2011). The results in Figure 4 demonstrate that human microbial taxa were present in indoor air at greater abundance than outdoors during times that the classroom was occupied. Taxa that are unique to human skin (*Propionibacterineae*, *Staphylococcus*, *Enterobacteriaceae*, and *Corynebacterineae*) were found in the indoor occupied setting at a total enrichment of 17% of the population, ranging from 31% in the largest (>9 μm) stage to 7.3% in the 1.1–2.1 μm size range. In the outdoor sample, only *Propionibacterineae* were observed in significant amounts, and the total enrichment of human skin, hair, and nostril microflora was 4.9%. Hence, the indoor airborne bacteria were enriched relative to outdoor airborne bacteria in these components of the human microbiome by a factor of 3.5 (17% divided by 4.9%). With the exception of *Staphylococcus* in the indoor occupied setting (which has potential skin and oral cavity sources), no bacterial taxa indicative of the oral cavity in humans were observed in the highly abundant taxa (Figure 4). Minor enrichments of *Pasteurellaceae* (0.4%) and *Fusobacterium* (0.2%) were found in the occupied setting and not in the outdoor aerosols, while low content of *Veillionella* was observed in both indoor (0.22%) and outdoor (0.4%) aerosols. Overall, the indoor–outdoor ratio of oral cavity bacterial abundance was two with *Staphylococcus* excluded and seven with *Staphylococcus* included.

Given that the emission rates of total 16S rDNA genomes for bacteria have been determined (Figure 3), relative abundance measures produced from pyrosequencing can then be used to estimate emission rates for specific taxa within a specific particle size range. Table 2 combines the bacterial emission rate data with the phylogenetic data to produce per-occupant emission rate estimates of human skin, hair, and nostril bacteria, as well as oral cavity bacteria in the studied classroom. These data reveal an emission rate of 5.9 × 10^6^ such bacteria per person-hour, with the dominant human bacterial emissions in the 3–4.7 and 4.7–9 μm size ranges. Human skin/hair/nostril emissions are significantly greater (∼10×) than those from the oral cavity, regardless of how *Staphylococcus* is categorized.

**Table 2:** Per person emission rates of human microflora for indoor occupied conditions in a university classroom^a^

	Skin/hair/nostrils microflora		Oral cavity microflora	
		
Particle aerodynamic diameter	*E* (10^6^ genomes/h/person)	Percent of total bacterial emissions (%)	*E* (10^6^ genomes/h/person)	Percent of total bacterial emissions (%)
0.4–1.1 μm	0.06	13	ND	ND
1.1–2.1 μm	0.14	7.3	0.03	1.8
2.1–3.3 μm^b^	–	–	–	–
3.3–4.7 μm	2.7	18	ND	ND
4.7–9 μm	2.0	15	0.5	4
>9 μm	0.53	32	ND	ND
Total	5.4	17	0.53	0.96

a Human skin microflora is comprised of the taxa *Propionibacterineae*, *Staphylococcus*, *Enterobacteriaceae*, and *Corynebacterineae*, while oral cavity microflora consists of *Pasteurellaceae*, *Fusobacterium*, and *Veillionella* spp. ND, not detected.

b PCR prior to sequencing failed on the particle size stage 2.1–3.3 μm.

## Discussion

The results of this study highlight two important concepts for understanding the sources of microbial aerosols in occupied indoor environments. First, human occupancy results in significant emissions of airborne particulate matter mass, bacterial genomes, and fungal equivalent genomes. Second, during occupancy, the bacterial phylogenetic analysis demonstrates a distinct indoor air signature of bacteria with associations to human skin, hair, and nostrils.

### Size-distributed emissions of particulate matter, bacteria, and fungi

Emissions of airborne bacteria and fungi in indoor environments have not been well characterized. However, studies based on surrogate measures for microorganisms (e.g., endotoxin or proteins) in multiple residences, as well as chamber and full-scale room resuspension rate estimates for allergens, all indicate that biological particles can be resuspended or directly shed during human occupancy (Chen and Hildemann, 2009; Clark, 1974; Fox et al., 2003; Goebes et al., 2011; Gomes et al., 2007; Raja et al., 2010). In the present study, the characterization of particle size distributions, the estimates of per person emissions rates, and the phylogenetic results revealed new insights into the major sources of fungal and bacterial indoor aerosols. The total mass emitted during occupancy was predominantly associated with large particles (>9 μm). This size-dependent increase is broadly consistent with previously observed increases in resuspension rate with increased particulate matter size (Ferro et al., 2004b; Thatcher and Layton, 1995).

In the case of fungi, the largest increase in concentration during occupancy was also observed on the largest impactor stage (>9 μm). Unicellular (2–5 μm) and multicellular (>10 μm) fungal spores are commonly present in indoor environments and thus provide for a potentially broad size distribution of fungal genomes. Multicellular fungal spores such as *Alternaria* spp. and *Epicoccum* spp. provide a source of larger fungal particles (Amend et al., 2010; Li and Kendrick, 1995) that can be preferentially resuspended during human activity. Under unoccupied conditions, one expects that these larger spores would be removed effectively by the filters in the building ventilation system, would penetrate through air leakage paths inefficiently, and would settle rapidly onto indoor surfaces compared to the smaller 2–5 μm unicellular fungi, such as *Aspergillus* spp. or *Cladosporium* spp., that are common indoors (Yamamoto et al., 2011). Relative to bacteria, the more modest overall increase in equivalent fungal genomes attributable to occupancy, especially below 9 μm aerodynamic diameter, is consistent with a recent study that demonstrated the roughly equivalent importance of both outdoor air concentrations and resuspension on influencing indoor air concentrations of *Aspergillus* spores (Goebes et al., 2011).

Bacterial genome emissions, in contrast, did not follow size distributions that would be expected from purely physical resuspension processes. While human occupancy led to a large total increase in bacterial genomes, the size distribution for bacterial genomes was not skewed toward the largest sizes, and increases in the largest impactor stage were substantially less than the average (for all sizes) bacterial genome increase (12× vs. 870×). The dominant size ranges for bacterial genomes were on the stage corresponding to 3.3–4.7 μm aerodynamic diameter. This size is larger than pure culture, single bacteria that have been previously screened for their aerodynamic diameter: *Serratia marcescens* 2.8 μm (Andersen impactor); *Bacillus subtilus* 2.7 μm (Andersen impactor); *Mycobacterium parafortuitum* 2.8 μm (Andersen impactor); *Pseudomonas fluorescens* 0.8 μm (aerodynamic size spectrometer) (Peccia et al., 2001; Willeke et al., 1996). Thus, we hypothesize that the organisms are commonly attached to each other or to other small biotic or abiotic particles. The wide dispersion of single taxa across multiple size ranges (Figure 4) also suggests that bacteria were mostly not present in particles as single cells. That indoor airborne bacteria may be found in aggregates has been previously reported by Lidwell et al. (1959). The size distribution observed in this study is consistent with recent outdoor measurements of bioaerosol particle concentration determined by an ultraviolet aerodynamic particle sizer that demonstrated a pronounced peak at 3.2 μm geometric mean diameter, which the authors attributed to unicellular fungal spores and agglomerated bacteria (Huffman et al., 2010).

The size distributions of bacterial genomes and fungal genomes clearly demonstrate that particulate matter mass is not a good surrogate for biological particles. During occupancy, the majority of total particle mass is larger than 9 μm in aerodynamic diameter; however, the dominant bacterial genome concentrations were between 3 and 5 μm, and the fungal genomes were broadly distributed. Other researchers have shown a lack of association between fungal spores (*Aspergillus* spp.) and particulate matter mass (Goebes et al., 2011). Focusing on bacteria in the 3- to 5-μm size range, such particles deposit relatively slowly to indoor surfaces. They would be removed efficiently by air filters common in mechanical ventilation systems (Nazaroff, 2004; Riley et al., 2002). However, indoor emissions could cause indoor exposures whenever occupants share indoor spaces. When inhaled, 3- to 5-μm particles have a high deposition efficiency in the human respiratory tract with substantial penetration to and deposition in the pulmonary (gas-exchange) region of the lung (Yeh et al., 1996).

The qPCR and phylogenetic-based analysis of particle size distributions for airborne fungi and bacteria is novel. A major advantage of these DNA-based methods is the complete responsiveness to bacterial and fungal organisms, regardless of the viability or culturability of the cells. Limitations in detecting environmental bacteria and fungi through culture-based methods are well documented (Amann et al., 1995; Peccia and Hernandez, 2006), as is the inactivation of bacteria during impaction or desiccation in impactors that are commonly used to provide size-resolved information (Stewart et al., 1995). Another important advantage of using DNA-based methods is the fact that agglomerated organisms are accurately counted, rather than determined as one colony-forming unit. However, when using universal primers for fungi, gene copies cannot be reliably converted to whole fungal spore counts owing to the variable number of 18S rDNA gene copies for different fungal species and the lack of consensus on the average operon gene copy number among all fungi. Moreover, the PCTE filters used were not coated to ensure successful cell extraction and downstream enzymatic-based PCR analysis. Particle bounce in the impactors could potentially bias the reported size distributions. However, the qualitatively good agreement between the OPC results and the impactor-based particle mass measurements suggests that any such bias, if present, was not strong.

### Bacterial diversity in the indoor occupied environment

A major contribution of the phylogenetic work reported here is to provide quantitative emission rates of bacteria that originate from human skin, hair, nostrils, and the oral cavity (Table 2). Such analysis demonstrates that the bacteria shed from humans can comprise a significant portion of the airborne bacteria to which people are exposed in common occupied spaces. Public health stresses the importance of human hygiene and specifically hand washing to prevent transmission of disease. While pathways for exposure to occupant-borne bacteria have mainly focused on touching fomites and on person-to-person direct contact, our results reinforce an understanding that humans may also be exposed to bioaerosol-borne microorganisms originating from other building occupants. These emissions might be produced by direct shedding into air or they might result from shedding followed by deposition and then resuspension. While the relative proportions of these mechanisms are unknown, there is evidence that both mechanisms may be significant. The increase in total particulate matter mass in larger sizes demonstrates a potentially significant role for resuspension. For direct emissions, previous literature indicates that the shedding of desquamated skin is a significant contributor to indoor aerosols (Clark, 1974; Fox et al., 2008; Noble et al., 1976). The emission rate of bacteria associated with skin shedding has been reported to range up to 6.8 × 10^5^ culturable colonies per person-hour (Mackintosh et al., 1978), which is ∼12% of the value for total human microbiota emissions reported here. We note that previous studies that focus on the bacteria shed from skin desquamation have demonstrated that the majority of skin bacteria are associated with particles larger than the 3–5 μm peak observed in our study (Fox et al., 2005; Mackintosh et al., 1978; Tham and Zuraimi, 2005). We note that the high enrichment of human microflora in larger-size bins of the indoor bioaerosol during occupancy indicates an increase in human bacterial emissions relative to total bacterial emissions in the larger size ranges (Table 2). The results provided here represent an early inquiry into the impacts of human occupancy on the type and character of biological aerosols present in indoor air. Linking occupant characteristics such as health status, clothing type, and individual activity is an important area of future research to produce directly applicable data on how specific characteristics of human occupancy impact exposure to etiological agents. Environmental parameters, such as season, building materials and furnishings, and geographic location also should be considered.

Limitations of this study included the restriction to one environment and that biological measurements were made from two indoor and two outdoor impactor samplers. Future investigations should extend this approach to multiple environments to determine the extent to which results such as these are broadly observed. An additional limitation emerges from uncertainty in interpreting data from the initial (largest) impactor stage. One aspect is the undefined upper size limit for particles collected on this stage. For analysis in this paper, we have assumed a sharp cut at 20 μm. Irrespective of this assumption, the mass and microbial genome concentration measurements for this upper size section are robust. However, the graphical presentation in Figure 1 would shift with a different upper size limit, with height changing so as to maintain a constant area for that section.

More significantly, converting from measured concentrations to emission factors (as depicted in Figure 3) depends on knowing the section-averaged particle deposition rate, *k*, which in turn depends on particle size. Overall, the literature lacks good empirical data on indoor deposition rates for particles larger than ∼ 10 μm. Furthermore, size-resolved deposition rates for bacteria and fungi have not been reported. The lack of microbe-specific deposition data may be a source of additional error owing to differences in shape and density from the olive-oil droplets studied by Thatcher et al. (2002). We based our analysis on empirical measurements (for 0.5- to 9-μm particles) reported by Thatcher et al. (2002) and extrapolated to cover the largest size section. If we had used gravitational settling as the basis for determining the loss-rate coefficient, then choosing 15 μm as the upper size limit would have reduced the loss-rate coefficient by 29%, whereas choosing 25 μm as the upper size limit would have increased it by 33%. For this range of aerodynamic diameters applied as the upper limit on the largest impactor size stage, there would be a ±20% corresponding uncertainty in emission rates. Errors in estimating the emission rates for other particle size sections are much smaller because of three factors: (i) well-defined upper size limits, (ii) stronger basis for the loss-rate coefficient, and (iii) less importance of the smaller loss-rate coefficients as compared to ventilation as a particle removal mechanism.

## Conclusions

Human occupancy in a university classroom was found to result in a large increase of indoor airborne particulate mass, bacterial genomes, and equivalent fungal genomes. The greatest increases in particle mass and fungal equivalent genomes were observed in the largest particle sizes, which is broadly consistent with expectations of progressively increasing influence of resuspension with increasing particle size plus the common presence of large (>10 μm) multicellular fungal spores in indoor environments. Enhanced bacterial concentrations during occupancy were predominantly found on particles with aerodynamic diameters in the 3- to 5-μm range. Emission rates per person were estimated for total particle mass, airborne bacterial genomes, and airborne fungal genomes. A significant proportion of the bacterial emissions (∼18%) were distinctively associated with the human microbiome. This evidence indicates that bioaerosols in occupied settings can be a significant vehicle for exposure to microorganisms shed by current or previous room occupants. The size-resolved emissions data represent an important step needed to develop effective models for predicting the fate of and exposure to microbial bioaerosols in indoor environments.

## Supporting Information

**Fig. S1** Size-resolved particle concentrations, vacant, 19 September 2009.

**Fig. S2** Size-resolved particle concentrations, vacant, 20 September 2009.

**Fig. S3** Size-resolved particle concentrations, vacant, 26 September 2009.

**Fig. S4** Size-resolved particle concentrations, vacant, 27 September 2009.

**Fig. S5** Size-resolved particle concentrations, occupied, 03 November 2009.

**Fig. S6** Size-resolved particle concentrations, occupied, 04 November 2009.

**Fig. S7** Size-resolved particle concentrations, occupied, 05 November 2009.

**Fig. S8** Size-resolved particle concentrations, occupied, 06 November 2009.

**Fig. S9**. Average (mass) particle sizedistribution based on optical particle counter (OPC) results.

**Fig. S10**. Relative abundance of dominant (top)and rare (bottom) bacterial phyla.

**Fig. S11** Heat map demonstrates the relative abundances of the 15 most common bacterial taxa in the indoor occupied and outdoor occupied samples.
