# Supplementary material for: Size-resolved emission rates of airborne bacteria and fungi in an occupied classroom
Source: Indoor Air. 2012 Aug;22(4):339–51. doi: 10.1111/j.1600-0668.2012.00769.x (PMC3437488; doi:10.1111/j.1600-0668.2012.00769.x)
Supplement: Supplementary file 1 [file ina0022-0339-SD1.doc]

Online Supporting Information for:

Size-resolved **emission rates of airborne bacteria and fungi in an occupied classroom**

**
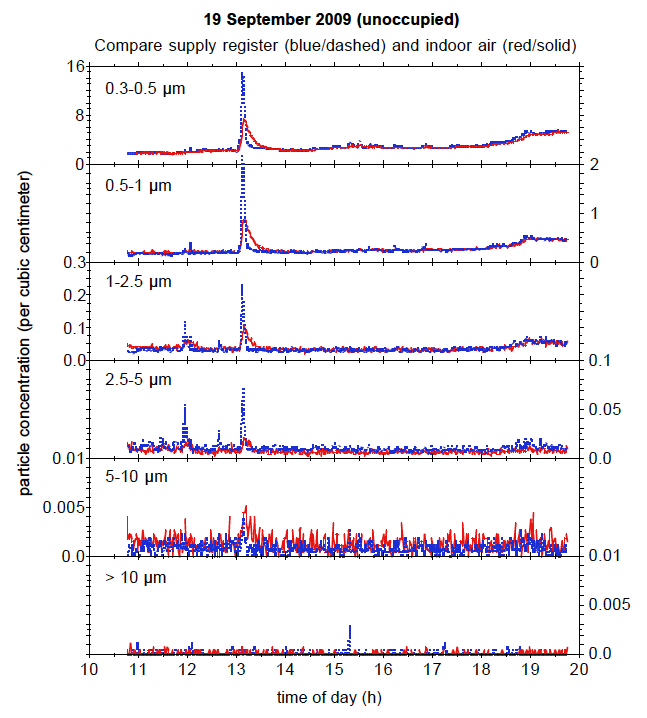
Fig. S1** Size-resolved particle concentrations, vacant, 19 September 2009. Impactor sampling pumps were operated for the entire duration shown.

**
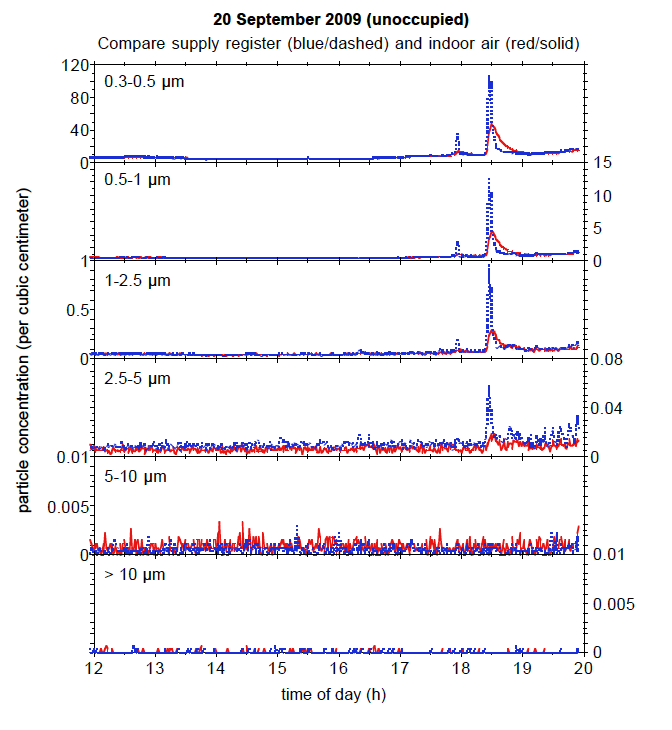
**

**Fig. S2** Size-resolved particle concentrations, vacant, 20 September 2009. Impactor sampling pumps were operated for the entire duration shown.

**
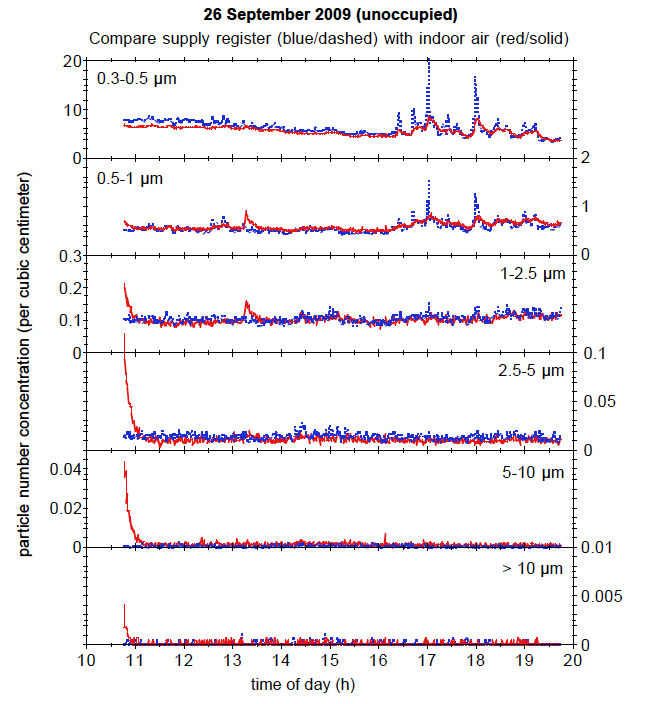
**

**Fig. S3** Size-resolved particle concentrations, vacant, 26 September 2009. Impactor sampling pumps were operated for the entire duration shown.

**
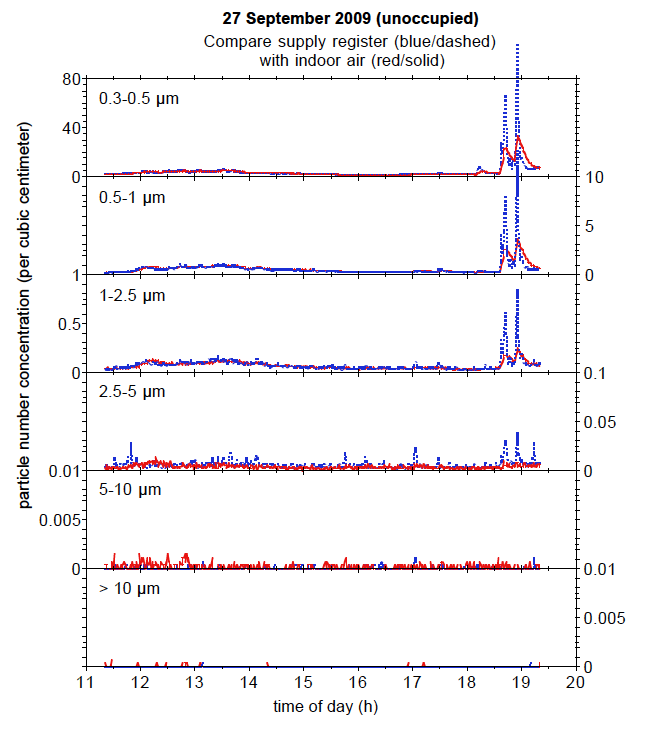
**

**Fig. S4** Size-resolved particle concentrations, vacant, 27 September 2009. Impactor sampling pumps were operated for the entire duration shown.

**
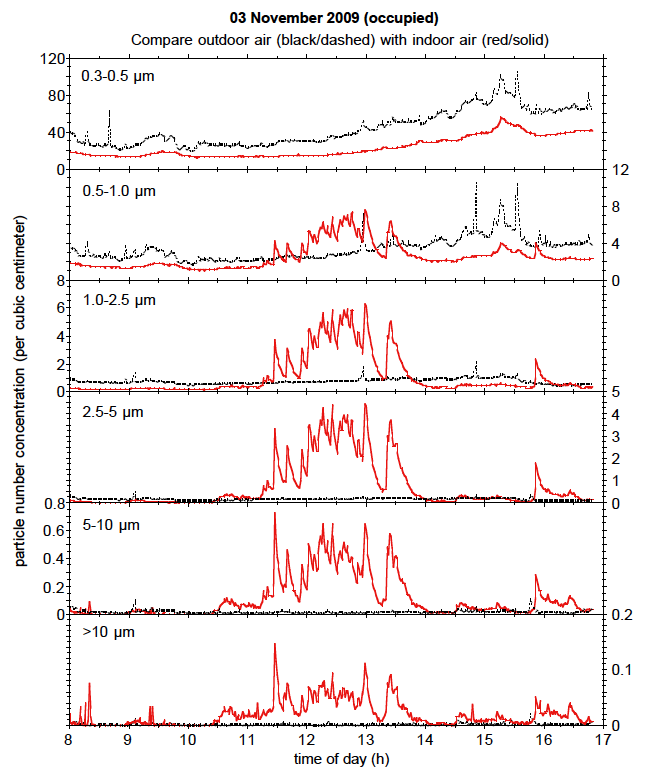
**

**Fig. S5** Size-resolved particle concentrations, occupied, 03 November 2009. Arrows indicate when impactor sampling pumps were in operation. Human occupancy levels were the following: 9-10:30, 0 people; 10:30-11:30, 11 people; 11:30-12:45, 12 people; 12:45-13:45, 3 people; 13:45 to 17:00, 0 people.

**
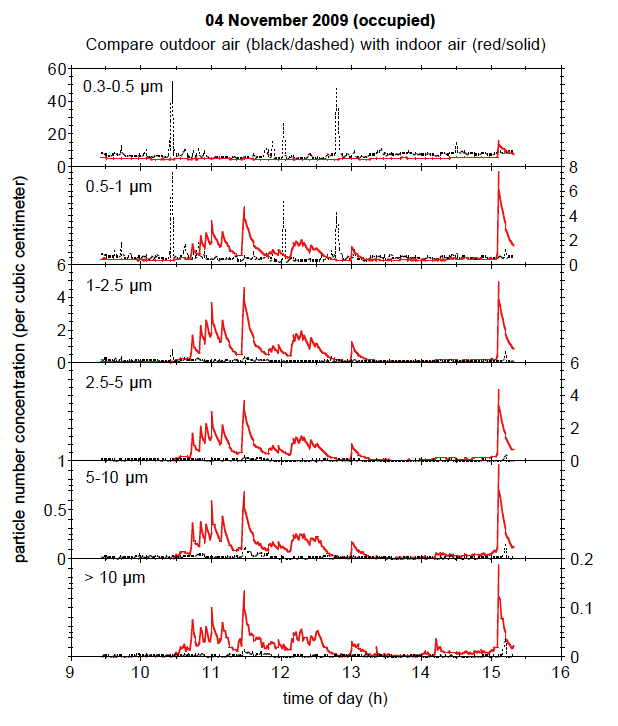
**

**Fig. S6** Size-resolved particle concentrations, occupied, 04 November 2009. Arrows indicate when impactor sampling pumps were in operation. Human occupancy levels were the following: 10:30-11:30, 14 people; 11:30-12:45, 12 people; 12:45-13:45, 2 people; 13:45-15:30, 0 people (peaks ~15:00 were caused by sample collection activity).

**
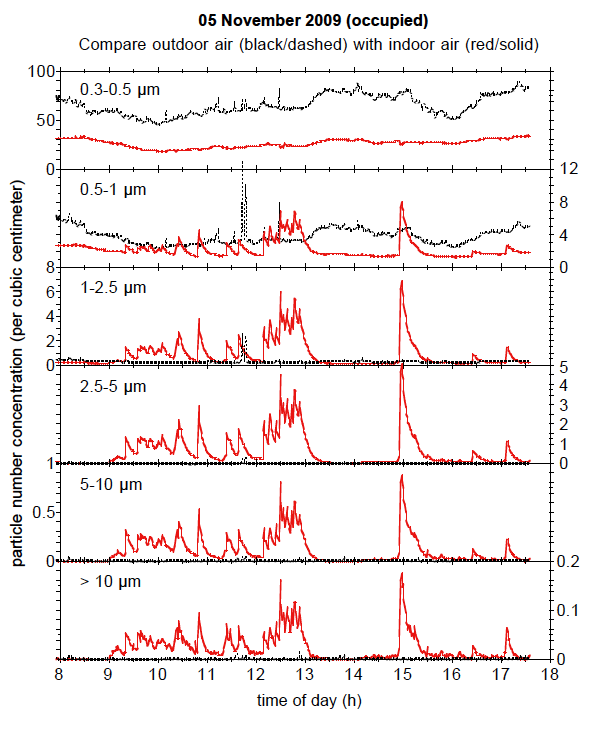
**

**Fig. S7** Size-resolved particle concentrations, occupied, 05 November 2009. Arrows indicate when impactor sampling pumps were in operation. Human occupancy levels were the following: 9-10:15, 4 people; 10:15-11:00, 14 people; 11:00-13:00, 13 people; 13:00-15:00, 0 people; 15:00 to 17:00, 0 people (peaks after 15:00 were cause by sample collection activity).

**
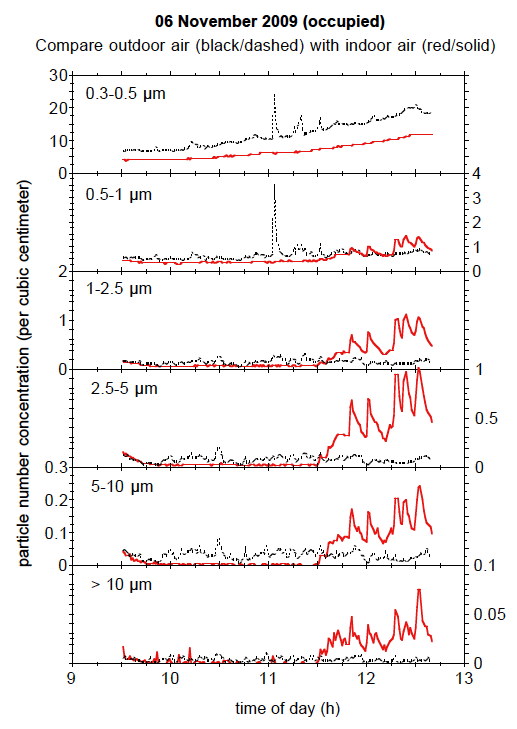
**

**Fig. S8** Size-resolved particle concentrations, occupied, 06 November 2009. Arrows indicate when impactor sampling pumps were in operation. Human occupancy levels were the following: 10:30 -11:30, 0 people; 11:30-12:40, 11 people.

**Fig. S9**. Average (mass) particle size distribution based on optical particle counter (OPC) results. A density of 2.5 g/cm^3^ was assumed in the conversion of particle volume to particle mass. An upper limit of 20 µm is assumed for the >10 μm OPC channel.

**Fig. S10**. Relative abundance of dominant (top) and rare (bottom) bacterial phyla. The dominant phyla are 98.1% of the sequences recovered from the indoor aerosols and 99.4% of the sequences recovered from the outdoor samples. Samples from all impactor stages are pooled in this analysis. The Cyanobacteria are dominated by chloroplast sequences from plant (Strephtophyta) material.

**Fig. S11.** Heat map demonstrates the relative abundances of the 15 most common bacterial taxa in the indoor occupied and outdoor occupied samples. Groups are classified to the highest taxonomic level to which they could be confidently assigned. Groups shown represent 57% of the occupied indoor air taxa and 68% of the outdoor air taxa. Taxa that are associated with the human microbiome are presented in bold text. The three separated taxa (*Veillionella*, *Fusobacterium*, and *Pasteurellaceae*) are low abundance and associated with the human oral cavity.
